# Evaluation of aphid resistance on different rose cultivars and transcriptome analysis in response to aphid infestation

**DOI:** 10.1186/s12864-024-10100-z

**Published:** 2024-03-04

**Authors:** Wenqi Dong, Lei Sun, Bo Jiao, Pu Zhao, Chunhong Ma, Junping Gao, Shuo Zhou

**Affiliations:** 1 Hebei Key Laboratory of Plant Genetic Engineering, Institute of Biotechnology and Food Science, Hebei Academy of Agriculture and Forestry Sciences, Shijiazhuang, 050051 China; 2 Department of Ornamental Horticulture, State Key Laboratory of Agrobiotechnology, Beijing Key Laboratory of Development and Quality Control of Ornamental Crops, College of Horticulture, China Agricultural University, Beijing, 100193 China

**Keywords:** Aphid, Rose, Jasmonic acid, WRKY transcription factor

## Abstract

**Background:**

The rose is one of the most important ornamental flowers in the world for its aesthetic beauty but can be attacked by many pests such as aphids. Aphid infestation causes tremendous damage on plant tissues leading to harmed petals and leaves. Rose cultivars express different levels of resistance to aphid infestation yet the information remains unclear. Not only that, studies about the transcriptional analysis on defending mechanisms against aphids in rose are limited so far.

**Results:**

In this study, the aphid resistance of 20 rose cultivars was evaluated, and they could be sorted into six levels based on the number ratio of aphids. And then, a transcriptome analysis was conducted after aphid infestation in one high resistance (R, Harmonie) and one highly susceptibility (S, Carefree Wonder) rose cultivar. In open environment the majority of rose cultivars had the highest aphid number at May 6th or May 15th in 2020 and the resistance to infestation could be classified into six levels. Differential expression analysis revealed that there were 1,626 upregulated and 767 downregulated genes in the R cultivar and 481 upregulated and 63 downregulated genes in the S cultivar after aphid infestation. Pathway enrichment analysis of the differentially expressed genes revealed that upregulated genes in R and S cultivars were both enriched in defense response, biosynthesis of secondary metabolites (phenylpropanoid, alkaloid, and flavonoid), carbohydrate metabolism (galactose, starch, and sucrose metabolism) and lipid processing (alpha-linolenic acid and linolenic acid metabolism) pathways. In the jasmonic acid metabolic pathway, linoleate 13S-lipoxygenase was specifically upregulated in the R cultivar, while genes encoding other crucial enzymes, allene oxide synthase, allene oxide cyclase, and 12-oxophytodienoate reductase were upregulated in both cultivars. Transcription factor analysis and transcription factor binding search showed that WRKY transcription factors play a pivotal role during aphid infestation in the R cultivar.

**Conclusions:**

Our study indicated the potential roles of jasmonic acid metabolism and WRKY transcription factors during aphid resistance in rose, providing clues for future research.

**Supplementary Information:**

The online version contains supplementary material available at 10.1186/s12864-024-10100-z.

## Background

As one of the most important ornamental flowers in the world, roses (*Rosa chinensis* L.) are attractive for their long flowering period, beautiful appearance, and a tremendous number of varieties; 30,000–35,000 cultivars are bred throughout the world [1]. However, due to their high carbohydrate and sugar content, roses are attacked by many pests, including rose aphid, *Macrosiphum rosae* (L.) [2].

There are 4,000 known aphid species worldwide, of which approximately 100 have successfully exploited the agricultural environment and pose a serious threat to crop production [3]. Aphids are phloem-feeding insects that cause direct and indirect damage to plants: direct damage is due to acquiring phloem nutrients necessary for plant growth, resulting in plant wilting and yield loss; and indirect damage is usually through honeydew excretion, saliva injection, and the transmission of plant fungal and viral pathogens [4]. Various insecticides have been used to inhibit aphid production, which may result in acquired resistance in pest species against these insecticides [5], as well as pollution to the ecological environment [6]. Therefore, the breeding of plant genotypes with strong resistance is a radical, yet inexpensive, and environmentally safe way to control aphids, which requires selection and evaluation of aphid resistance on different rose cultivars, and a full understanding of the mechanism of plant resistance to aphids.

There are three types of resistance to aphids, namely antixenosis, which is rejection of a plant when a choice is possible; antibiosis, which causes adverse effects on the phytophage viability during feeding; and tolerance [7]. Different types of resistance are usually found to be controlled by different genes [8], and several gene loci were simultaneously involved in antixenosis and antibiosis in some crop genotypes [9, 10], resulting in a complex mechanism of resistance to aphids.

To elucidate the changes in gene expression in response to aphids on a genomic scale, many “-omics” research studies have been conducted in several crops, including wheat (*Triticum aestivum* L.) [11, 12], maize (*Zea mays* L.) [13], celery (*Apium graveolens* cv. Dulce) [14], *Brassica juncea* [15], and soybean (*Glycine max* L. Merr) [16]. These studies have identified many genes involved in the plant response to aphids, including those involved in signal transduction; transcriptional regulation; reactive oxygen species; protein synthesis, modification, and degradation; maintenance of cell structure and homeostasis; and secondary metabolism [17]. However, only a few genes have been cloned and confirmed to provide resistance to aphids. Two nucleotide-binding-site leucine-rich repeat (NBS-LLR) proteins, *Mi-1* and virus aphid transmission (*Vat*), confer resistance to *Macrosiphum euphorbiae* in tomato [18] and *Aphis gossypii* in melon [19], respectively. In *Arabidopsis*, a small heat shock-like protein, sieve element-lining chaperone 1 (*SLI1*), confers resistance to the tobacco aphid *M. persicae nicotianae*, the cabbage aphid *Brevicoryne brassicae*, and the cabbage whitefly *Aleyrodes proletella* [20, 21], implying a broad-spectrum resistance to phloem-feeding insects.

It has been shown that phytohormone, jasmonic acid (JA), and salicylic acid (SA) signal transduction pathways were associated with aphid resistance in plants. The JA synthesis-related genes *LOX*, *AOS*, and *AOC* were significantly upregulated in aphid-feeding sites in wheat [22], while exogenous application of JA effectively decreased aphid reproduction in cucumber leaves [23]. Additionally, SA content in the leaves of barley plant increased with aphid infestation [24], and exogenous SA improved the resistance of wheat to the grain aphid [25]. However, the molecular mechanism of resistance to aphids in plants is yet to be clarified.

In this study, 20 rose cultivars, which were commonly used for ornamental flowers in North China, were selected for evaluation of aphid resistance. And then, a comparative transcriptomic analysis after aphid infestation in aphid-resistant (R) and -susceptible (S) rose cultivars was carried out to identify the aphid resistance on different rose cultivars, to discovery aphid-resistance–related genes in rose and to characterize the potential roles of phytohormone and transcription factors (TFs) during aphid resistance in rose.

## Results

### Evaluation of aphid resistance on 20 rose cultivars

Generally, the number of aphids increased firstly and then decreased from April 29th to May 29th in most of rose cultivars, except for tengbenyueji and Gräfin von Hardenberg. The number of aphids on these two cultivars decreased continuously, with a highest aphid number on April 29th. Additionally, the density of aphids of 8 rose cultivars, including Dortmund, Garden Fun, Jayne Austin, Agnes Schilliger, Shizuku, Mary Ann, Harmonie, and Parkdirektor Riggers, peaked on May 6th; the density of aphids of 9 rose cultivars, including Ramukan, Carefree wonder, My Choice, Caramella, Souvenir de Louis, Louise Odier, Fancy Ruffle, Highgrove, and Mozart, peaked on May 15th; and one rose cultivar, bel canto, maintained the highest density of aphids on May 22nd (Table 1).

**Table 1:** Resistance levels to aphids of 20 rose cultivars

Rose Cultivars	The Number of Aphids					Number Ratio of Aphids	Resistance Level
	**Apr. 9th**	**May. 6th**	**May. 15th**	**May. 22th**	**May. 29th**		
Dortmund	102.00	116.67	45.67	20.87	5.27	1.552	HS
Ramukan	27.47	46.60	53.67	22.73	1.07	0.810	S
Carefree Wonder	32.20	47.40	290.33	265.13	52.00	3.672	HS
Garden Fun	48.93	56.40	53.33	22.67	1.13	0.975	S
Jayne Austin	45.33	85.80	68.33	13.53	1.33	1.146	MS
Agnes Schilliger	45.47	83.20	72.60	18.53	0.87	1.179	MS
My Choice	11.27	17.87	22.60	10.47	0.47	0.335	MR
Caramella	4.93	14.13	25.47	12.53	4.47	0.329	MR
Shizuku	17.50	27.07	7.53	3.87	1.60	0.301	MR
tengbenyueji	276.67	266.40	46.80	20.80	10.00	3.317	HS
Gräfin von Hardenberg	91.93	89.60	57.93	28.47	0.73	1.436	HS
Souvenir de Louis	15.07	27.33	30.27	3.13	0.20	0.406	MS
Mary Ann	51.67	75.87	27.93	21.93	1.93	0.958	S
Louise Odier	4.07	3.60	7.20	5.93	0.33	0.113	HR
bel canto	1.60	5.40	2.53	5.93	0.47	0.085	HR
Harmonie	2.07	5.13	2.27	0.73	0.20	0.056	HR
Parkdirektor Riggers	20.13	61.53	8.87	9.60	0.07	0.536	R
Fancy Ruffle	3.07	12.27	17.80	6.80	0.00	0.213	HR
Highgrove	10.07	20.93	25.40	22.13	1.53	0.428	MR
Mozart	10.40	132.80	145.00	107.07	2.53	2.126	HS

Based on the number ratio of aphids, the aphid resistance of 20 rose cultivars could be sorted into six levels, in which 5 cultivars, Dortmund, Carefree Wonder, tengbenyueji, Gräfin von Hardenberg, and Mozart were high susceptibility to aphid (RL > 1.25); 3 cultivars, Jayne Austin, Agnes Schilliger, and Souvenir de Louis were moderate susceptibility to aphid (RL = 1.01–1.25); 3 cultivars, Ramukan, Garden Fun, and Mary Ann were susceptibility to aphid (RL = 0.76–1.00); 1 cultivar, Parkdirektor Riggers was resistance to aphid (RL = 0.51–0.75); 4 cultivars, My Choice, Caramella, Shizuku, and Highgrove were moderate resistance to aphid (RL = 0.26–0.50); 4 cultivars, Louise Odier, bel canto, Harmonie, and Fancy Ruffle were high resistance to aphid (RL = 0.01–0.25) (Table 1).

### Transcriptome profiles of *Rosa chinensis* treated with aphids

To investigate the underlying mechanisms of aphid resistance in rose, two cultivars exhibiting diverse levels of resistance to aphid infestation, the R cultivar (Harmonie) for aphid resistance and the S cultivar (Carefree Wonder) for aphid susceptibility, were chosen and infested by aphids. Deep RNA-seq sequencing of the R and S cultivars was performed, with and without infestation, and each with three biological repeats. A total of 549.96 million filtered high-quality reads from 12 libraries (Q30 ranged from 95.1% to 95.6%) were obtained. The basic sequencing statistics are shown in Table S1. Previously, two research groups had successfully conducted full genome sequencing of the homozygous cultivar ‘Old Blush’. Clean reads were aligned to both reference genomes, *Rosa chinensis* Whole Genome v1.0 (OBDH-1.0) and *Rosa chinensis* Old Blush homozygous Genome v2.0 (RchiOBHm-V2), and the alignment rates are shown in Table 2. The overall alignment rate was slightly higher in OBDH-1.0 than in RchiOBHm-V2, with an average value of 86.26 and thus was used as the reference genome. RNA-seq read coverage showed that the results met the characteristics of normal RNA sequencing (Fig. S1).

**Table 2:** Alignment Rate comparison of two reference genomes

Sample	Total pairs	RchiOBHm-V2		OBDH-1.0	
		Total unpaired reads	Overall alignment rate	Total unpaired reads	Overall alignment rate
RC_1	22858915	8091862	87.07%	7493628	87.91%
RC_2	23637813	8529840	86.76%	7910258	87.60%
RC_3	20949180	7906024	86.01%	7365110	86.84%
RT_1	23829302	9391070	85.01%	9117610	85.32%
RT_2	23542911	9241142	85.78%	9108868	85.92%
RT_3	21001268	8398804	84.87%	8065864	85.34%
SC_1	22946976	9660454	84.96%	8958872	86.01%
SC_2	22972798	8730880	85.43%	7956048	86.57%
SC_3	22870614	9597474	85.12%	8795614	86.34%
ST_1	23790608	10315194	84.19%	9412998	85.54%
ST_2	23384605	9592846	84.96%	8762290	86.22%
ST_3	23196030	9943186	84.22%	9074692	85.53%

### Expression levels of genes in the R and S cultivars

The expression level and density of all genes were normalized as FPKM (Fig. S2A, B; Table S2). Using the FPKM value in the samples > 1 as a threshold, a total of 32,930 expressed genes filtered from 44,481 mapped genes were identified.

**Fig. 1:**
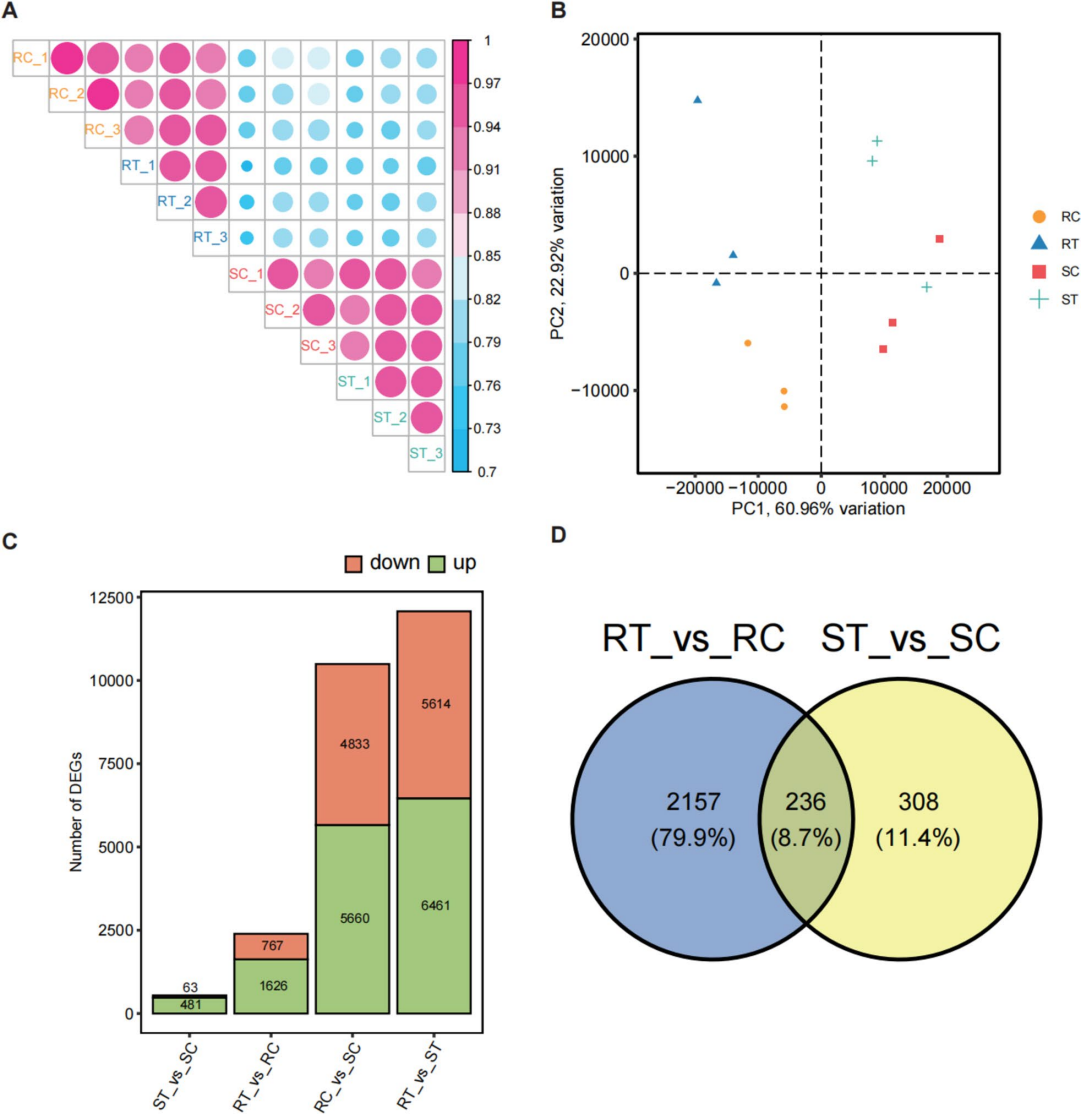
Global view of gene expression profiles and changes of two rose cultivars under aphid infestation. **A** Spearman correlation coefficient (SCC) of gene expression profiles between samples. RC, RT, SC, and ST are samples of two cultivars (R and S) with (T) or without (C) aphid infestation. The size and color of each circle indicate the coefficient value between each sample. **B** Principal component analysis (PCA) of samples distinguished by different colors with three biological repeats. **C** Number of differentially expressed genes (DEGs) compared between different samples. **D** Cross-comparison Venn diagram showing the number of DEGs following aphid infestation in the R and S cultivars

Principal component analysis (PCA) and Spearman correlation coefficient (SCC) were used to gain a global overview of the transcriptomic differences (Fig. 1A, B). Correlations among samples in the same cultivar showed relatively higher values than those between two cultivars with or without the aphid infestation. PCA showed that cultivars R and S were separated by PC1 (60.96% variation). Overall, these results indicated that the R and S cultivars showed distinct responses to aphid infestation.

### Transcriptome changes in the R and S cultivars after aphid infestation

DEGs were detected using an adjusted *P*-value (padj) < 0.05 and |log_2_ foldchange|> 1 as thresholds. When infested by aphids, there were 1,626 upregulated and 767 downregulated genes in the R cultivar and 481 upregulated and 63 downregulated genes in the S cultivar, while a large number of DEGs (> 10,000) were also found between the R and S cultivars whether or not they were infested by aphids (Fig. 1C). A Venn diagram showed common or uniquely regulated genes between the two cultivars infested by aphids, and only 8.7% of DEGs were common (Fig. 1D). When infested by aphids, the log_2_ foldchange of DEGs was mainly found in the interval [1, 2] (Fig. S2C, D). Volcano plots showed gene IDs with restricted cut-offs (|log2 foldchange|> 2, − log10padj > 9) (Fig. S2E, F). These results revealed an interspecific difference between the R and S cultivars. The mechanisms confronting aphids may be partially identical between the two cultivars.

### Expression clusters by k-means

**Fig. 2:**
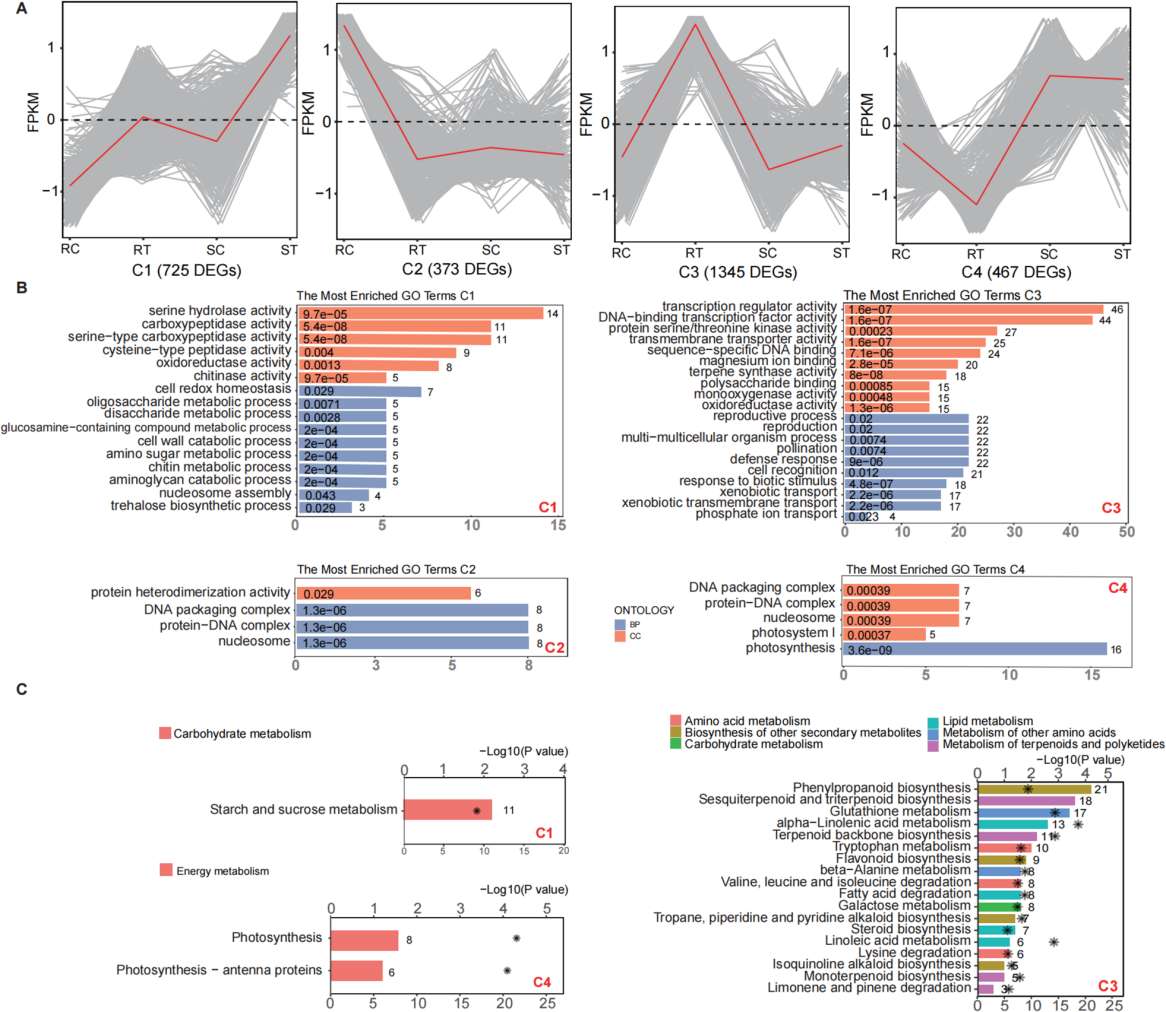
Cluster analysis of DEGs based on the k-means method. **A** Four clusters (C1–C4) based on the k-means algorithm. The Y-axis stands for scaled FPKM. Gene expression profiles in the line plots are shown in gray, and the mean values are shown in red for each cluster. RC, RT, SC, and ST are samples of one high resistance (R, Harmonie) and one highly susceptibility (S, Carefree Wonder) rose cultivar with (T) or without (C) aphid infestation. **B** Enriched GO terms of genes in the four clusters. Terms were ranked by the number of genes. **C** KEGG pathway enrichment analysis of three clusters. *P*-value levels are indicated as − log10 (*P* value), and the values are represented by the asterisks near the bar. No pathways were found enriched in Cluster C2

All DEGs between the R and S cultivars following aphid infestation were further categorized into four clusters based on k-means clustering analysis (Fig. 2A). The optimal k value (4) was determined by the elbow method (Fig. S3). Of the four clusters, C1 (725) and C3 (1,345) represented genes that were upregulated with aphid infestation, whereas C2 (373) and C4 (467) represented genes that were downregulated. In upregulated clusters, genes in C3 were specifically highly expressed in the R cultivar, and genes in C1 were specifically highly expressed in the S cultivar.

### GO and KEGG enrichment of DEGs and expression clusters

**Fig. 3:**
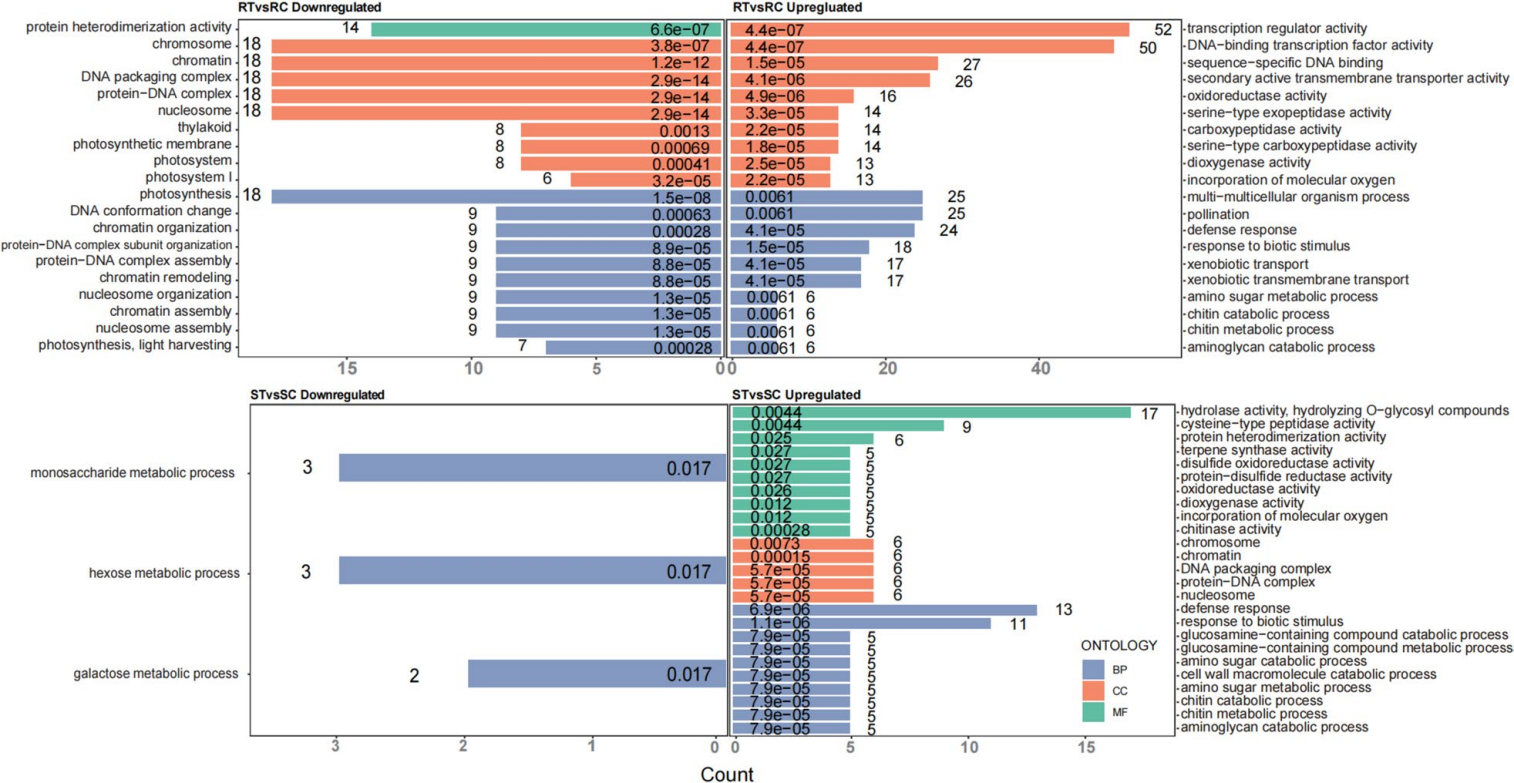
Bar plots showing the most enriched GO terms in the two cultivars after aphid infestation. Upregulated and downregulated DEGs in the two cultivars were analyzed by GO enrichment separately. RC, RT, SC, and ST are samples of one high resistance (R, Harmonie) and one highly susceptibility (S, Carefree Wonder) rose cultivar with (T) or without (C) aphid infestation. GO terms were subclassified into three categories distinguished by different colors: biological processes (BP), cellular components (CC), and molecular function (MF). Terms were primarily ranked by the number of genes participating in the relevant pathway. Values at the bottom of each bar represent the adjusted *P*-value (padj) attributed to the enrichment of the relevant pathway

The significant DEGs were then annotated based on functional GO from GDR using Fisher's precision probability test (Table S3). Using GO enrichment analysis, DEGs were divided into three major enrichment categories: molecular functions (MF), cellular components (CC), and biological processes (BP). At most 10 enriched terms of each category were listed in bar plots. There were fewer enriched terms in MF compared to CC and BP in the R cultivar. Terms enriched in CC showed that upregulated genes in the R cultivars were enriched in transcription regulator activity, DNA-binding transcription factor activity, and sequence-specific DNA binding, while the terms enriched in BP revealed that upregulated genes in the R and S cultivars were both enriched in defense response, response to biotic stimulus, and sugar, chitin, and aminoglycan metabolism. The analysis also showed that downregulated genes in the R cultivar were enriched in photosynthesis, while several metabolic processes of carbohydrates were suppressed in the S cultivar (Fig. 3).

**Fig. 4:**
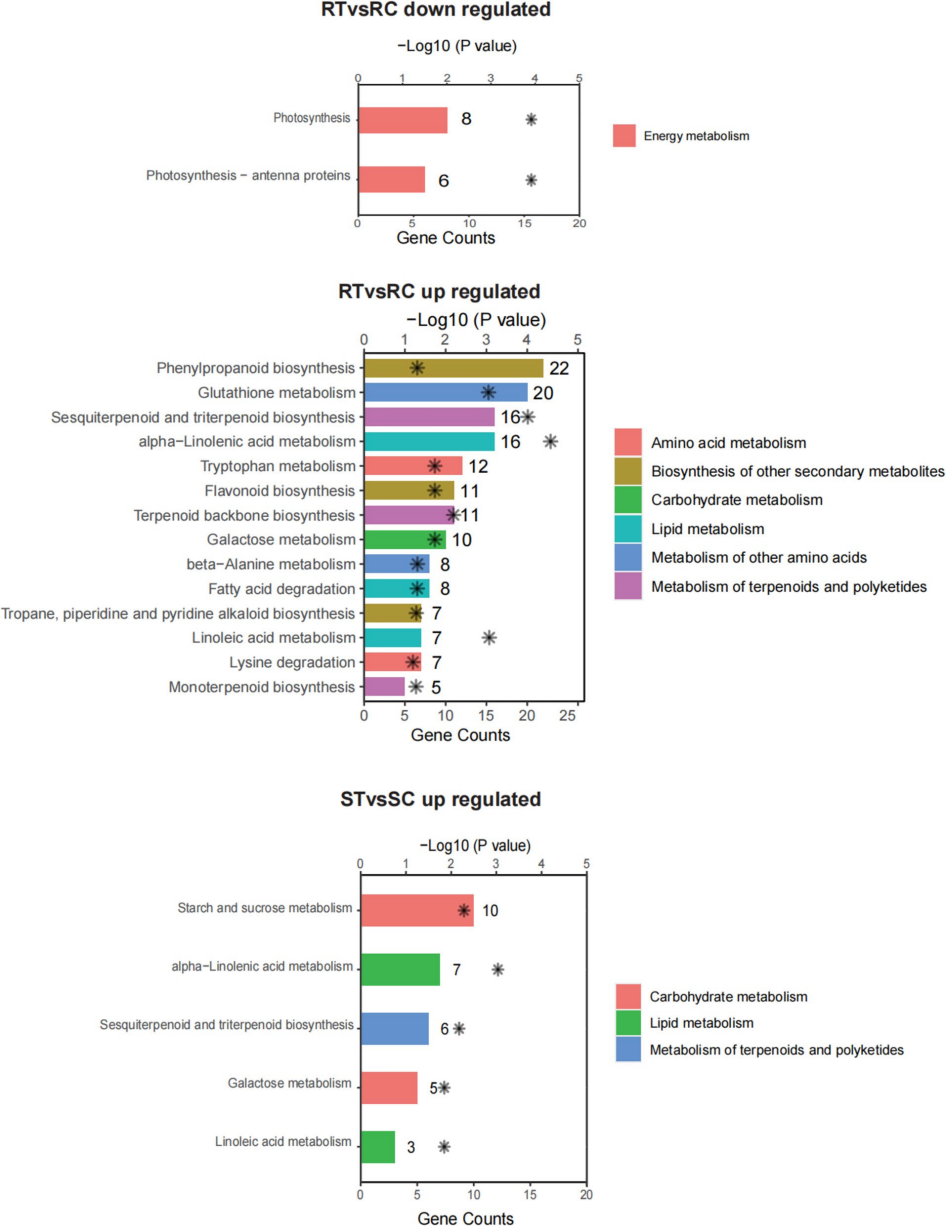
Distribution of KEGG pathways in the two cultivars after aphid infestation. Upregulated and downregulated DEGs in the two cultivars were analyzed by KEGG enrichment separately. RC, RT, SC, and ST are samples of one high resistance (R, Harmonie) and one highly susceptibility (S, Carefree Wonder) rose cultivar with (T) or without (C) aphid infestation. Enriched terms are visualized by bar plots with *P*-value levels indicated as − log_10_ (*P* value). The values are represented by the asterisks near the bar

KEGG enrichment analysis were further conducted using a local KofamKOALA database (Table S4). DEGs of two cultivars were assigned to significant (*p* < 0.05) KEGG pathways (Fig. 4). This showed that biosynthesis of secondary metabolites (phenylpropanoid, alkaloid, and flavonoid), carbohydrate metabolism genes (galactose, starch, and sucrose metabolism), and lipid processing (alpha-linolenic acid and linolenic acid metabolism) were enriched in upregulated genes in both the R and S cultivars, while there were no enriched terms of downregulated genes in the S cultivar.

Next, we conducted GO and KEGG enrichment analyses for clusters based on gene expression patterns to differentiate genes linked with aphid resistance more clearly (Fig. 2B, C). Cluster C1 consisted of genes with the highest expression after aphid infestation in the S cultivar. Enrichment analysis showed that this cluster was enriched in chitin metabolic processes, cell wall catabolic processes, and carbohydrate metabolism. Cluster C3 consisted of genes with the highest expression in the R cultivar, in which lipid metabolism and biosynthesis of secondary metabolites, such as phenylpropanoid and flavonoid biosynthesis, were enriched. Since anabolism of JA and its derivatives is part of alpha-linolenic metabolism and phenylpropanoid is a precursor to SA, these results indicated that plant hormones, especially JA and SA, may play crucial roles in aphid resistance, and that the divergent expression levels of their related metabolism between the R and S cultivars may explain the distinct levels of resistance. Compared to genes in Cluster C3, genes in Cluster C1 may represent background mechanisms of pest infestations, such as cell wall and chitin metabolic process.

In the alpha-linolenic metabolism pathway depicted by the R package pathview (Fig. S4), genes encoding linoleate 13S-lipoxygenase (LOX, EC1.13.11.12) were specifically upregulated in the R cultivar, while genes encoding other crucial enzymes for generating the basic structure of JA, including allene oxide synthase (AOS, EC4.2.1.92), allene oxide cyclase (AOC, EC5.3.99.6), and 12-oxophytodienoate reductase (OPR, EC1.3.1.42), were upregulated in both the R and S cultivars. It is noteworthy that the expression level of the gene encoding JA carboxyl methyltransferase (JMT, EC 21.1.141) was higher in the S cultivar than in the R cultivar. Taken together, the results showed that JA biosynthesis-related genes were induced by aphid infestation, implying the potential roles of JA in aphid resistance.

### TFs identified in the response to aphid infestation

To determine the transcription factors involved in the aphid infestation response in each cultivar and cluster, all DEGs were compared to the iTAK database based on HMMER. Of the 1,745 TFs identified by the iTAK database, 159, 30, 31, 23, 88, and 26 predicted TFs from 49 distinct families were present in RT vs RC, ST vs SC, C1, C2, C3, and C4, accounting for 6.01%, 6.17%, 6.54%, 5.57%, 6.64%, and 5.51% of each subset, respectively. We found that APETALA2/ethylene-responsive factor (AP2/ERF), basic/helix-loop-helix (bHLH), MYB, NAC, and WRKY were more abundant in C3 and RTvsRC. Significantly enriched analysis using Fisher’s exact test (padj < 0.05) was then performed, and we found that WRKY and AP2/ERF were enriched in both RTvsRC and C3.

**Fig. 5:**
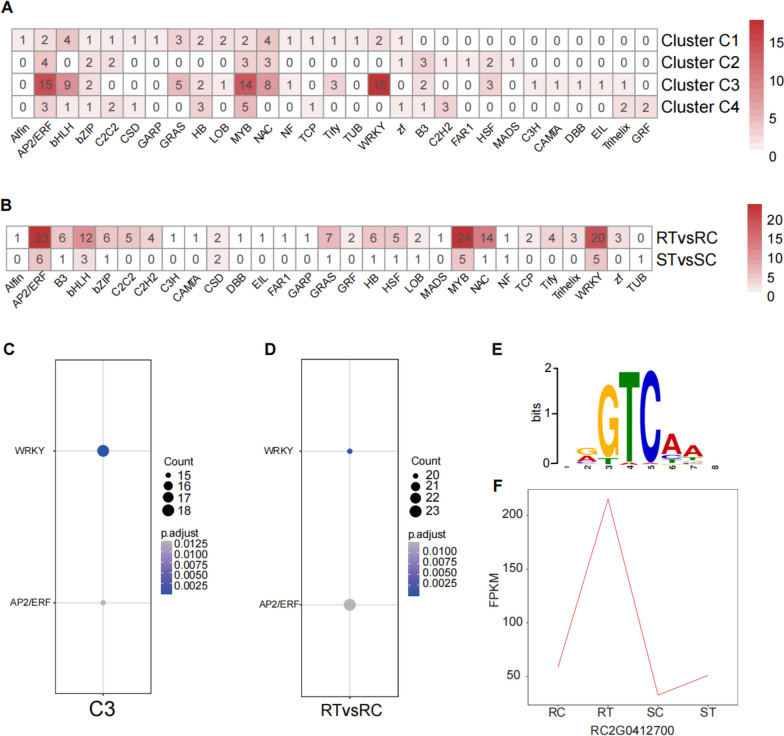
The identification of transcription factors (TFs) and the abundant binding sites (TFBS). **A**, **B** Distribution of TF families in the four clusters and two cultivars. RC, RT, SC, and ST are samples of one high resistance (R, Harmonie) and one highly susceptibility (S, Carefree Wonder) rose cultivar with (T) or without (C) aphid infestation. The color represents the number of genes in each TF family. **C**, **D** Significantly overrepresented TF families within Cluster C3 and the R cultivar in response to aphid infestation. **E** The seqlogo of abundant TFBS (TFmatrixID_0445) in Cluster C3. **F** The expression pattern of gene RC2G0412700 based on FPKM

Considering the expression pattern and GO analysis of DEGs in Cluster C3, C3 may explain the different resistance levels between the R and S cultivars. Therefore, the upstream 1,000 base pairs of DEGs in C3 were used as queries and analyzed using the PlantPAN database. Using transcription factor binding search (TFBS), five TF binding sites were identified in more than 1,000 DEG promoters. They were bZIP, bHLH, C2H2, WRKY40, and NAC (Table S5). We noticed that the description of WRKY (TFmatrixID_0445, “tgGTCAAt”) was related to the pathogen-induced transcription factor, and the expression pattern of its homologous gene in rose (RC2G0412700) was the same as that in C3 (Fig. 5). Considering its biochemical function in biotic stress, RC2G0412700 may play a vital role in pest resistance to aphids.

### DEGs involved in phytohormone metabolism during aphid infestation

**Fig. 6:**
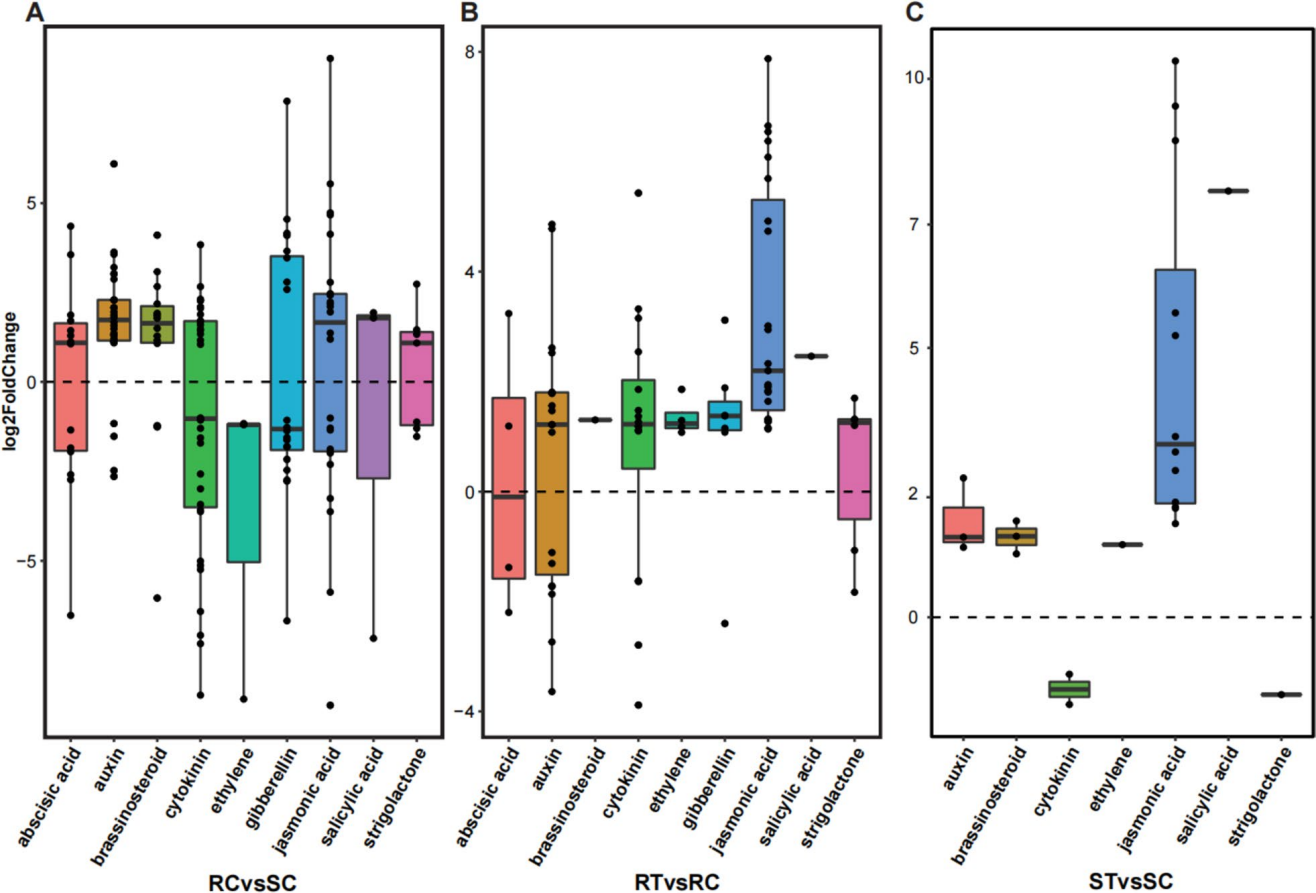
Expression patterns of genes involved in different phytohormone metabolism pathways. **A** The differences in phytohormone metabolism between the two cultivars in a normal state. **B**, **C** Dynamic changes in phytohormone metabolism in the R and S cultivars infested by aphids. RC, RT, SC, and ST are samples of one high resistance (R, Harmonie) and one highly susceptibility (S, Carefree Wonder) rose cultivar with (T) or without (C) aphid infestation

Phytohormones play indispensable roles in orchestrating biotic plant defenses. Proteins expressed by genes involved in phytohormone metabolic pathways, including abscisic acid (ABA), auxin (IAA), brassinosteroid (BR), cytokinin (CK), ethylene (ET), gibberellin (GA), JA, SA, and strigolactone (SL) were analyzed through MapMan together with the PlantCyc databases. We found that the expression levels of genes associated with the phytohormone metabolic pathways in the R and S cultivars were different in normal states. When infested with aphids, genes involved in the JA and SA pathways were significantly upregulated in both the R and S cultivars (Fig. 6). Gene expression related to phytohormones identified by MapMan is shown in a heatmap (Fig. S5).

### PPI network among the DEGs

PPI analysis of DEGs involved in the hormone process was performed to identify cross-talk among different hormones. The network was built using Cytoscape and only genes with relatively strong physical interactions are shown (Fig. S6). Ten genes with the highest ranking are listed (Table 3). There were genes participating in various hormone metabolic processes, suggesting a mixed hormonal regulation of the plant biotic defense response.

**Table 3:** Top10 genes with highest degree in PPI (Hormone)

GID	description
RC2G0361000	systemin receptor SR160
RC6G0486000	allene oxide synthase 1, chloroplastic
RC3G0212400	ethylene-insensitive protein 2
RC5G0431800	indole-3-acetaldehyde oxidase
RC7G0341900	abscisic acid 8'-hydroxylase CYP707A2
RC2G0102800	auxin response factor 7
RC1G0530300	gibberellin 20 oxidase 1
RC7G0016500	probable auxin efflux carrier component 1c
RC4G0088000	auxin response factor 19
RC4G0345700	histidine kinase 3

We also analyzed PPI relationships of DEGs in C3 to obtain a better understanding of the molecular mechanism of aphid resistance (Fig. S6). The building process was the same as described above, and 10 genes with the highest ranking were identified (Table 4). We found three WRKY transcription factors (RC4G0344000, RC6G0452500, and RC2G0412700) and a probable linoleate 9S-lipoxygenase 5 related to linolenic acid metabolism.

**Table 4:** Top10 genes with highest degree in PPI (Cluster C3)

GID	description
RC2G0102100	protein TIFY 10a
RC5G0530300	mitogen-activated protein kinase 3
RC4G0344000	probable WRKY transcription factor 75
RC3G0338600	calmodulin-binding protein 60 D
RC3G0353500	probable linoleate 9S-lipoxygenase 5
RC7G0296400	transcription factor MYC2
RC6G0452500	probable WRKY transcription factor 40
RC6G0394600	calmodulin-like protein 8
RC2G0412700	probable WRKY transcription factor 40
RC4G0311300	transcription factor MYB108

### Gene Expression Validation by qRT-PCR

**Fig. 7:**
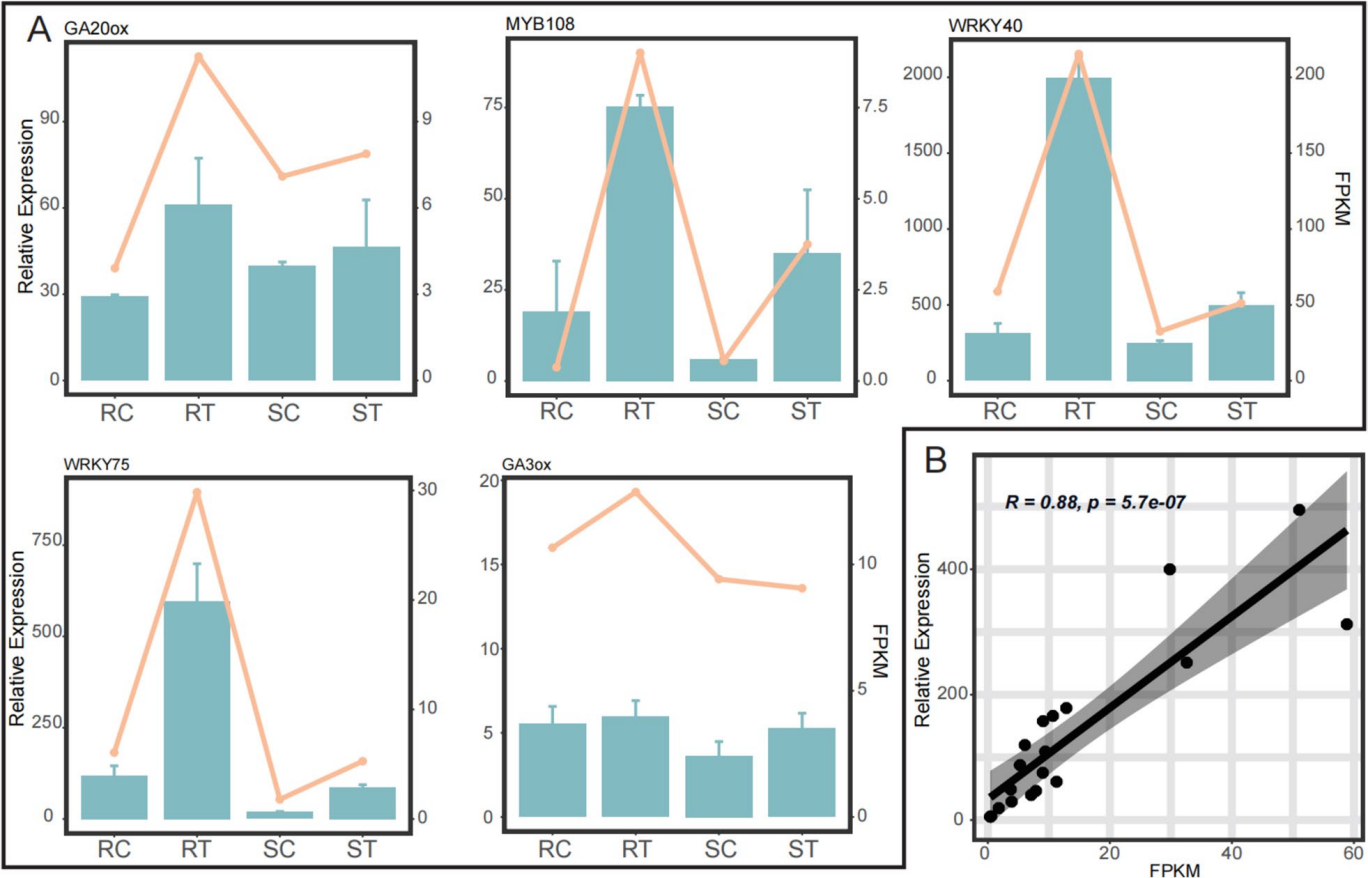
Verification of RNA-seq by qRT-PCR and the correlation analysis. **A** Expression patterns of 5 selected genes measured using the 2^−ΔΔCT^ method. The histograms in cyan represented RT-PCR results with Error bars showing the means ± SEM. The orange lines represented the average values of FPKM for each sample. RC, RT, SC, and ST are samples of one high resistance (R, Harmonie) and one highly susceptibility (S, Carefree Wonder) rose cultivar with (T) or without (C) aphid infestation. **B** Correlation analysis for 5 selected genes between RNA-Seq and qRT**-**PCR

Quantitative real-time PCR was conducted to further validate the reliability of the RNA-seq data with 4 clustered expression patterns as well as the PPI analysis results. So, we selected 4 DEGs which have relative higher degree in PPI analysis (*GA20ox*, *WRKY75*, *WRKY40* and *MYB108*) and 1 random non-differential expression gene (*GA30ox*) for verification. The results of qRT**-**PCR were basically consistent with the RNA-seq results (Fig. 7A). *GA20ox*, *WRKY75*, *WRKY40* and *MYB108* were dramatically induced in R cultivar, implying these genes may be involved in aphid resistance in rose.

We further analyzed the correlation between RNA-Seq and qRT**-**PCR results. The high correlation (*R* = 0.88, *p* = 5.7e-07) supports the reliability of the transcriptome results (Fig. 7B).

## Discussion

As one of most popular ornamental plant, rose are usually attacked by aphids, which can reduce the flower growth and quality. In this study, it indicated that the rose cultivars were vary in resistance to aphids (Table 1), consistent with other report on 10 rose cultivars [26]. The highest density of aphids was found on May 6th or May 15th on most of rose cultivars, which may be useful for developing an integrated pest management in rose plants. Additionally, 4 rose cultivars, Harmonie, bel canto, Louise Odier and Fancy Ruffle were found to be high resistance to aphid, which can be important germplasms for aphid resistance breeding in rose.

RNA-seq is a standard method for measuring and comparing the levels of gene expression in a wide variety of species and conditions [27]. Comparative transcriptome analyses focusing on aphid infestations were conducted in sorghum [28], wheat [29], soybean [16], maize [13], and rose [30], in which hundreds of genes were found to be differentially expressed after aphid infestation. In this study, aphid infestation triggered the expression of many genes (Fig. 1A). Interestingly, more DEGs after aphid infestation can be observed in R cultivar than S cultivar, implying a stronger response to aphids can be triggered at the transcriptional level in the R cultivar, which may contribute to high resistance.

TFs play vital roles in regulating the gene expression involved in both biotic and abiotic defense responses [31]. In our study, GO analysis showed that C3 and DEGs upregulated in R cultivar were enriched in transcription regulator activity, DNA-binding transcription factor activity, and sequence-specific DNA binding (Figs. 2, 3). It suggested that transcriptional regulation of downstream genes was very important for rose to resist aphids.

As one of the largest families of TFs in plants, WRKY transcription factors modulate many plant processes [32], including defense signaling [33]. Previous reports suggested that WRKY TFs played pivotal roles in aphid resistance. In tomato, the *SlWRKY70* transcript level was inducible in response to aphid infestation, and silencing *SlWRKY70* attenuated *Mi-1*-mediated resistance against aphids [34]. The overexpression of *CmWRKY48* inhibited the aphid population growth capacity in chrysanthemum [35]. Importantly, *RlWRKY10* and *RlWRKY14* in rose (*Rosa longicuspis*) were positive regulators in aphid resistance [36]. However, *TaWRKY53* in wheat [37], *CmWRKY53* in chrysanthemum [38], and *AtWRKY22* in *Arabidopsis* [39] negatively regulated the resistance to aphids. In this study, it can be observed that WRKY TFs were enriched in both RTvsRC and C3 (Fig. 5), and the binding sites of WRKY can be found in the promoter of DEGs in R cultivar after aphid infestation (Table S5), implying the potential roles of WRKY TFs in aphid resistance in rose, which may be the regulators for transcriptional regulation of downstream genes.

The phytohormone JA contributed to plant defense against biotic stresses, including insect attacks [40]. JA and its cyclopentanone derivatives are also involved in inducing a defense against aphids. For example, exogenous application of JA or JA-Ile significantly enhanced the aphid resistance in potato [41], cucumber [23], and soybean [42], and JA biosynthesis was enhanced after aphid infestation [42]. In our results, KEGG analysis showed that DEGs upregulated in R cultivar were enriched in alpha-Linolenic acid metabolism (Fig. 4), which was a key step for JA biosynthesis. It can be also observed that genes encoding crucial enzymes for generating the basic structure of JA, including *13-LOX* that was specifically upregulated in R cultivar, and *AOS*, *AOC*, and *OPR* that were upregulated in both the R and S cultivars (Fig. S4), suggested the important role of JA in aphid resistance in rose, in which LOX may be a key regulator. However, although the JA level was transiently increased at the early stages of aphid feeding on an aphid-resistant sorghum cultivar, exogenous application of JA promoted improved aphid feeding and colonization [43], which indicated a dichotomous role of JA in aphid resistance. Interestingly, the expression levels of *LOX* genes, which catalyze the first committed step of JA biosynthesis, are generally regulated by WRKY TFs [44, 45], which were up-regulated by aphid infestation in this study (Fig. 5; Table 4). The precise regulatory mechanism of JA in aphid resistance in rose need more research to reveal.

SA also plays a crucial role in resistance to biotic stresses. It has shown that the functions of SA in resistance to aphids varied in plant species. SA can enhance defense response to Russian wheat aphid (RWA) in wheat [46], reduced plant damage and suppressed sugarcane aphid (SCA) population growth and fecundity in sorghum [47]. However, overexpression of Armet, an effector protein, can induce a fourfold increase in SA accumulation and enhance the plants’ resistance to bacterial pathogen *Pseudomonas syringae*, but had no detectable adverse effects on aphid survival or reproduction [48]. In this study, KEGG analysis showed that DEGs upregulated in R cultivar were enriched in phenylpropanoid biosynthesis (Fig. 4), which is related with SA biosynthesis, while induced *PAL* expression is associated with SA accumulation in plants [49, 50]. Considering the complexity between SA and aphid resistance, the function of SA in resistance to aphids in rose needs further confirmation.

## Conclusions

In summary, our study evaluated the aphid resistance of 20 rose cultivars, which could be sorted into six levels based on the number ratio of aphids. Transcriptome analysis in response to aphid infestation characterized several genes triggered by aphid infestation, which may be likely regulated by the WRKY transcription factor, and associated with JA or/and SA biosynthesis or signal transduction. Our work is of great significance for screening of aphid-resistant rose germplasm and the functional identification of aphid-resistant genes.

## Materials and methods

### Evaluation of aphid resistance on different rose cultivars

The aphid resistance on 20 rose cultivars were evaluated, including Dortmund, Ramukan, Carefree wonder, Garden Fun, Jayne Austin, Agnes Schilliger, My Choice, Caramella, Shizuku, tengbenyueji, Gräfin von Hardenberg, Souvenir de Louis, Mary Ann, Louise Odier, bel canto, Harmonie, Parkdirektor, Riggers Fancy Ruffle, Highgrove, and Mozart. In October 2018, the scions of the 20 tested rose cultivars with robust and consistent growth state were selected for cutting propagation and rooting in a greenhouse. On April 30, 2019, they were planted in an open environment at our field experiment station in Baoding City, Hebei Province, China. One rose cultivar was planted with an area of 1.8 m × 1.4 m, row spacing of 45 cm and plant spacing of 20 cm, in a random block arrangement with three repeats. Conventional water and fertilizer were used without pesticide during the experiment period.

The number of aphids was counted in an open environment on April 29th, May 6th, May 15th, May 22nd, and May 29th in 2020. Ten rose plants were selected randomly for every cultivar, and the number of aphids was counted within 10 cm of stem tip. The resistance level to aphid of one rose cultivar was represented using a number ratio of aphids, in which the resistance of rose cultivars to aphids could be sorted into six levels (Table 5).

**Table 5:** The resistance levels based on number ratio of aphids

Resistance level (RL)	Number Ratio of Aphids
High Resistance (HR)	< 0.25
Moderate Resistance (MR)	0.26–0.50
Resistance (R)	0.51–0.75
Susceptibility (S)	0.76–1.00
Moderate Susceptibility (MS)	1.01–1.25
High Susceptibility (HS)	> 1.25

Number Ratio of aphids = the number of aphids in one rose cultivar/the average number of aphids in all rose cultivars.

### Aphid infestation for transcriptome analysis

Based on the identification of aphid resistance, two rose cultivars, Harmonie (high resistance to aphid) and Carefree Wonder (high susceptibility to aphid) were planted in a greenhouse and selected for transcriptome analysis. The rose plants were challenged with 20 aphids. Leaf tissues were collected after 72 h from aphid-treated and control plants (RC, control plants for aphid-resistant cultivar; RT, aphid-treated plants for aphid-resistant cultivar; SC, control plants for aphid-susceptible cultivar; ST, aphid-treated plants for aphid-susceptible cultivar).

### RNA extraction, Illumina library construction, and sequencing

Samples were ground in separate RNase-free mortars filled with liquid nitrogen. Total RNA was extracted using an RNAprep Pure Plant kit (Tiangen, Beijing, China) according to the manufacturer’s instructions. An RNA Nano 6000 assay kit, part of the Bioanalyzer 2100 system (Agilent Technologies, CA, USA), was used to assess the RNA integrity. Then, mRNA was extracted by VAHTS mRNA capture beads (Vazyme Biotech, Nanjing, China) following the manufacturer’s protocol. To generate the sequencing libraries, an NEBNext Ultra RNA library prep kit (NEB, USA) was used. After PCR product purification and library quality assessment, RNA sequencing was subsequently performed on an Illumina Novaseq platform (Illumina, USA) by Novogene Corporation (Beijing, China), and 150 bp paired-end reads were generated.

### Quality control, trimming, and mapping of reads

Sequenced raw reads in a fastq format were filtered with FASTP v0.23 [51] to remove low-quality reads and reads containing adapter and N bases using the default parameters. Paired-end clean reads were then mapped to the reference genome *Rosa chinensis* Genome v1.0 (assessed on February 2018) [52] with the parameters “–new-summary –dta” after building the genome index by HISAT2 v2.2.1 [53]. SAM files were converted into BAM files using SAMtools v1.10 [54], and Qualimap v2.2.1 [55] was used to evaluate the sequencing alignment data. To count the read numbers in each gene model shown in the gff file, the R package FeatureCounts v1.5.0 [56] was used, and the expression level of each gene was normalized as fragments per kilobase of transcript per million mapped reads (FPKM). To remove low-expression genes, genes were filtered with the threshold FPKM deg > 1. Visualized results of principal component analysis (PCA) using the R package PCAtools v2.8.0 [57] and correlation analysis based on the Spearman correlation coefficient method were used for quality analysis. The R package factoextra v1.0.7 [58] was used to analyze the hierarchical relationship by building a dendrogram.

### Identification and functional annotation of DEGs

Differentially expressed genes (DEGs) between any two samples were identified by the R package DESeq2 v1.20.0 [59]. The *P*-value was adjusted by the Benjamini and Hochberg method, and a *P*-value (padj) < 0.05 and |log_2_ foldchange|> 1 were used as the cut-off criteria for screening significant DEGs. An analysis of common and unique DEGs between different samples was conducted by visualizing results acquired from the R package VennDiagram [60]. A k-means cluster analysis was performed based on the R package factoextra [58]. The k number was chosen using the elbow method for the most optimal cluster number. Functional annotations of DEGs were generated based on Mercator4 [61] with MapMan and Plant Metabolic Network metabolic pathway databases (https://plantcyc.org/).

### GO and KEGG pathway enrichment analysis

Gene Ontology (GO) terms analyzed by InterProScan [62] and Kyoto Encyclopedia of Genes and Genomes (KEGG) pathways analyzed through the KEGG automatic annotation server (KAAS) [63] of *Rosa chinensis* based on its transcripts are available on Genome Database for Rosaceae (GDR) (https://www.rosaceae.org/) [64]. After building the R package OrgDB of *Rosa chinensis* using the R package AnnotationForge [65], GO and KEGG pathway enrichment analyses of DEGs were implemented by the R package clusterProfiler with padj < 0.05 as the threshold [66].

### TF identification and PPI analysis

Plant TFs were predicted through iTAK v1.5 [67], a TF database based on PlnTFDB [68] and PlantTFDB [69]. Full-length protein sequences were used in iTAK as queries to acquire the composition of each TF family in the *Rosa chinensis* genome, against which different DEG groups were aligned. Significant enrichment of TF families was analyzed using the R package clusterProfiler [66] with *P*-value < 0.05 set as the cut-off.

Information on rose proteins in the STRING database (https://cn.string-db.org/) was obtained and then used as a BLAST db. After sequence alignment to the local BLAST database by BLAST [70], STRING v11.5 [71] was used to predict protein–protein interactions (PPIs), and the resulting network was visualized through Cytoscape v3.9 [72].

### Validation of transcriptome results using quantitative real-time PCR

The Quantitative Real-time PCR was carried out on a 7500 Fast Real-Time PCR System (Applied Biosystems, Foster, CA, USA) using ChamQ SYBR qPCR Master Mix (Vazyme, Nanjing, China) with the following procedure: 95 °C for 30 s, followed by 40 cycles of 95 °C for 10 s, 60 °C for 30 s, and a melt curve stage of 95 °C for 15 s, 60 °C for 1 min, and 95 °C for 30 s. RcActin was used as an internal control for its consistent expression. The relative expression level of genes was calculated by the method of 2^−ΔΔCT^ [73]. Each treatment has triplicate biological replicates which was performed with three technical repeats. Gene-specific primers were listed in Table S6. The correlation analysis between RNA-Seq and qPCR results was conducted under R based on the Pearson method. The plot was drawn by R package ggpubr.

### Supplementary Information

**Supplementary Material 1. **

**Supplementary Material 2.**

**Supplementary Material 3. **

**Supplementary Material 4. **

**Supplementary Material 5. **

**Supplementary Material 6. **

**Supplementary Material 7. **

**Supplementary Material 8. **

**Supplementary Material 9. **

**Supplementary Material 10. **

**Supplementary Material 11. **

**Supplementary Material 12. **

## Data Availability

The transcriptomic data presented in this study are openly available on the National Center for Biotechnology Information (NCBI) BioProject PRJNA968003. The experimental materials, 20 rose cultivars were obtained from and planted by the Institute of Biotechnology and Food Science, Hebei Academy of Agriculture and Forestry Sciences (HAAFS). All databases in this study are available to the public.
