# Supplementary material for: Evaluation of aphid resistance on different rose cultivars and transcriptome analysis in response to aphid infestation
Source: BMC Genomics. 2024 Mar 4;25:232. doi: 10.1186/s12864-024-10100-z (PMC10910744; doi:10.1186/s12864-024-10100-z)
Supplement: Supplementary file 1 — Supplementary Material 1. [file 12864_2024_10100_MOESM1_ESM.pdf]

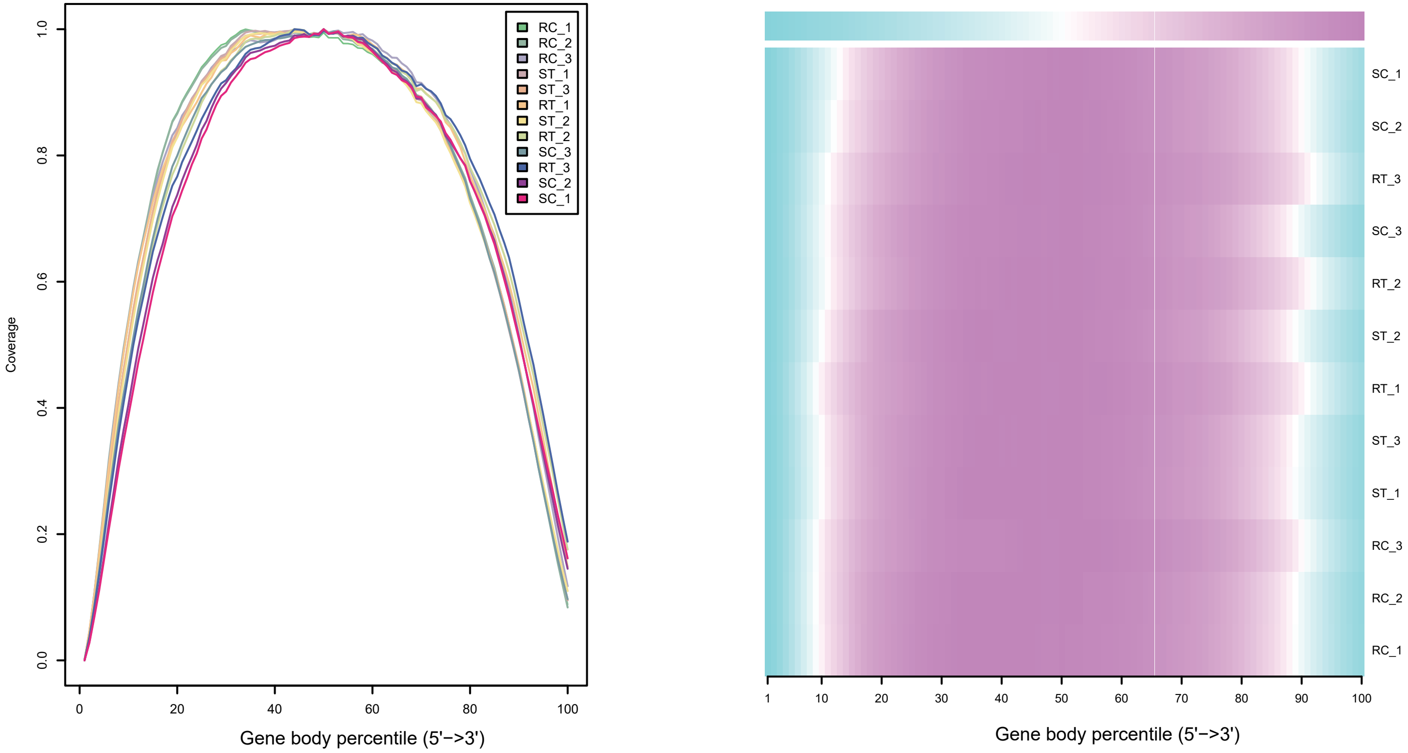

Figure S1. RNA-seq quality control by the R package RSeQC
RNA-seq read coverage over the gene body shown as (A) a line graph and (B) a heatmap.
