# Supplementary material for: Evaluation of aphid resistance on different rose cultivars and transcriptome analysis in response to aphid infestation
Source: BMC Genomics. 2024 Mar 4;25:232. doi: 10.1186/s12864-024-10100-z (PMC10910744; doi:10.1186/s12864-024-10100-z)
Supplement: Supplementary file 2 — Supplementary Material 2. [file 12864_2024_10100_MOESM2_ESM.pdf]

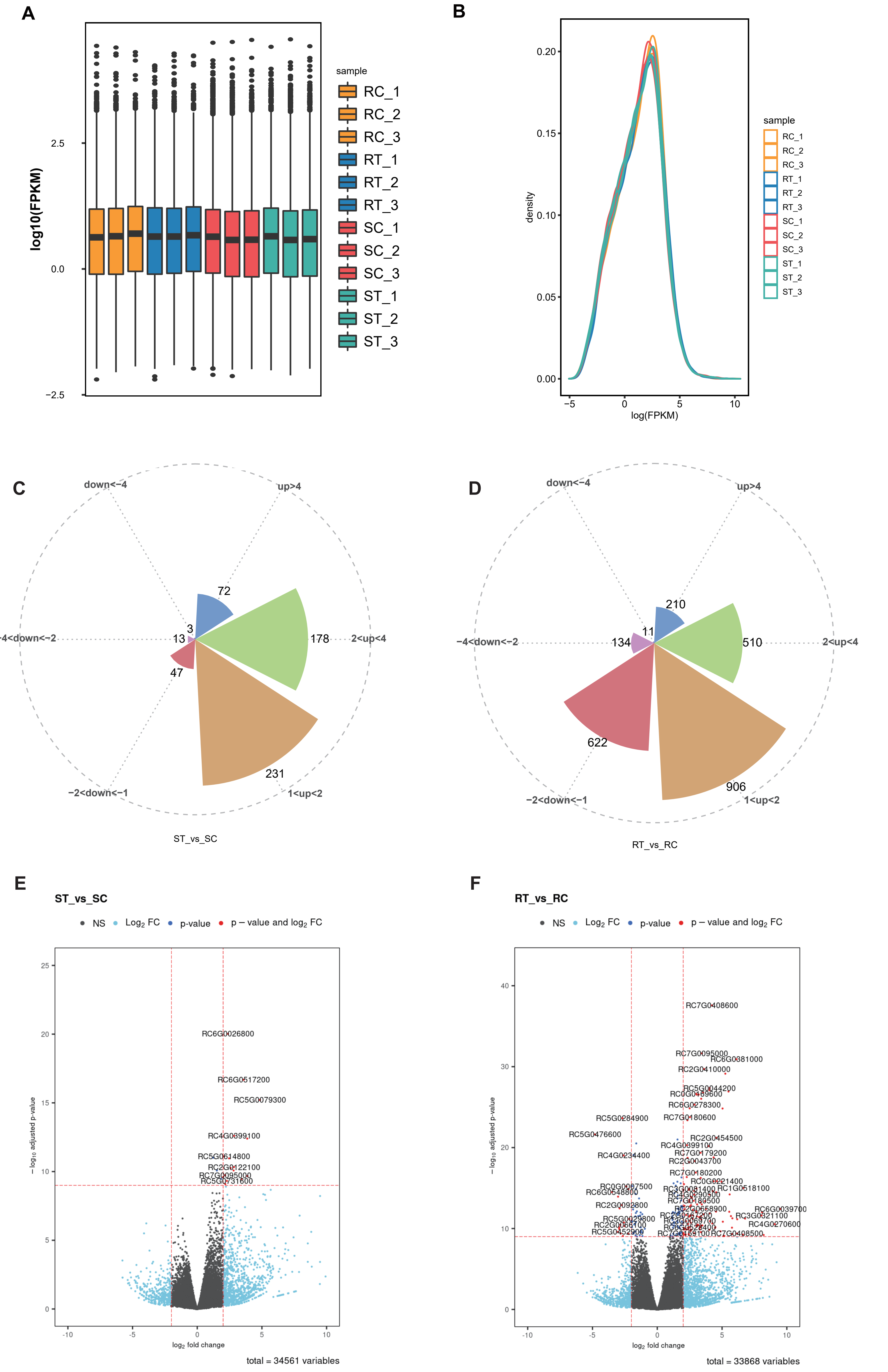

Figure S2. The distribution of epressed genes and DEGs for rose samples under different treatment

(A) Box plot showing expressed genes in each sample. RC, RT, SC, and ST are samples of two cultivars (R and S) with (T) or without (C) aphid infestation. Central black line represents median values; bounds of the box are 25% and 75% of the total genes. (B) Density of gene expression. (C) Number of differentially expressed genes (DEGs) compared between different samples. (D) Cross-comparison Venn diagram showing the number of DEGs following aphid infestation in the R and S cultivars. (C, D) Pie plots showing distribution of DEGs in the S cultivar and R cultivar. Values in the pies indicate the number of DEGs in each interval. (E, F) Volcano plots showing DEGs in the two cultivars. Genes meeting the criteria ($|\log_2$ foldchange$| > 2$, $-\log_{10}$ padj > 9, red dashed lines) are listed.
