# Supplementary material for: Evaluation of aphid resistance on different rose cultivars and transcriptome analysis in response to aphid infestation
Source: BMC Genomics. 2024 Mar 4;25:232. doi: 10.1186/s12864-024-10100-z (PMC10910744; doi:10.1186/s12864-024-10100-z)
Supplement: Supplementary file 3 — Supplementary Material 3. [file 12864_2024_10100_MOESM3_ESM.pdf]

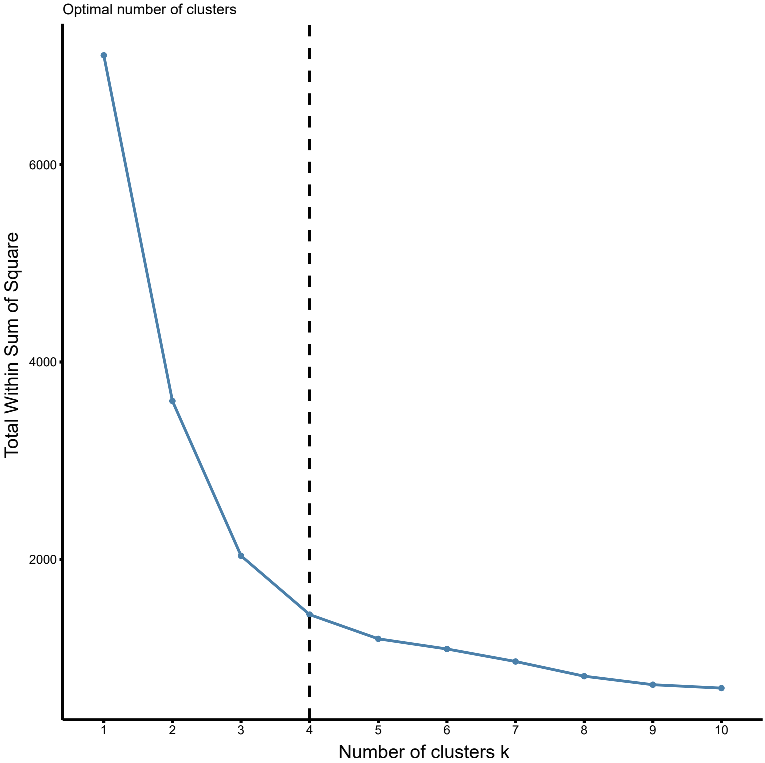

Figure S3. Selection of an optimal number of clusters for k-means clustering approach.
The optimal k number is demarcated by the black dashed line.
