# Supplementary material for: Evaluation of aphid resistance on different rose cultivars and transcriptome analysis in response to aphid infestation
Source: BMC Genomics. 2024 Mar 4;25:232. doi: 10.1186/s12864-024-10100-z (PMC10910744; doi:10.1186/s12864-024-10100-z)
Supplement: Supplementary file 4 — Supplementary Material 4. [file 12864_2024_10100_MOESM4_ESM.pdf]

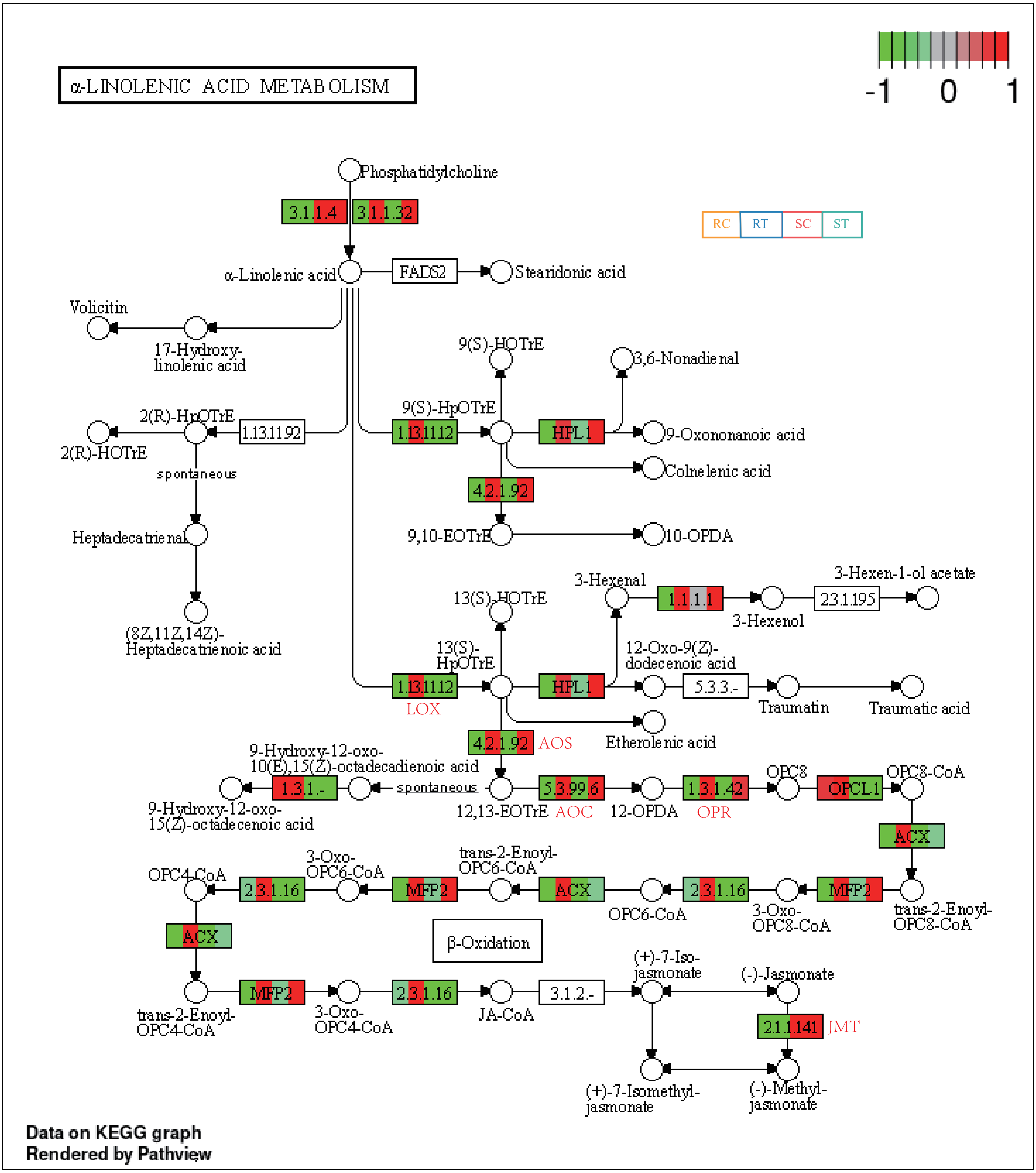

Figure S4. Expression patterns of genes involved in alpha Linolenic metabolism pathway. The gene expression was displayed based on scaled FPKM values.Red and green represent the elevation and decrease of corresponding gene expression, respectively. Each colored box represents RC, RT, SC, ST with three biological repeats.
