# Supplementary figures and images for: Evaluation of aphid resistance on different rose cultivars and transcriptome analysis in response to aphid infestation
Source: BMC Genomics. 2024 Mar 4;25:232. doi: 10.1186/s12864-024-10100-z (PMC10910744; doi:10.1186/s12864-024-10100-z)

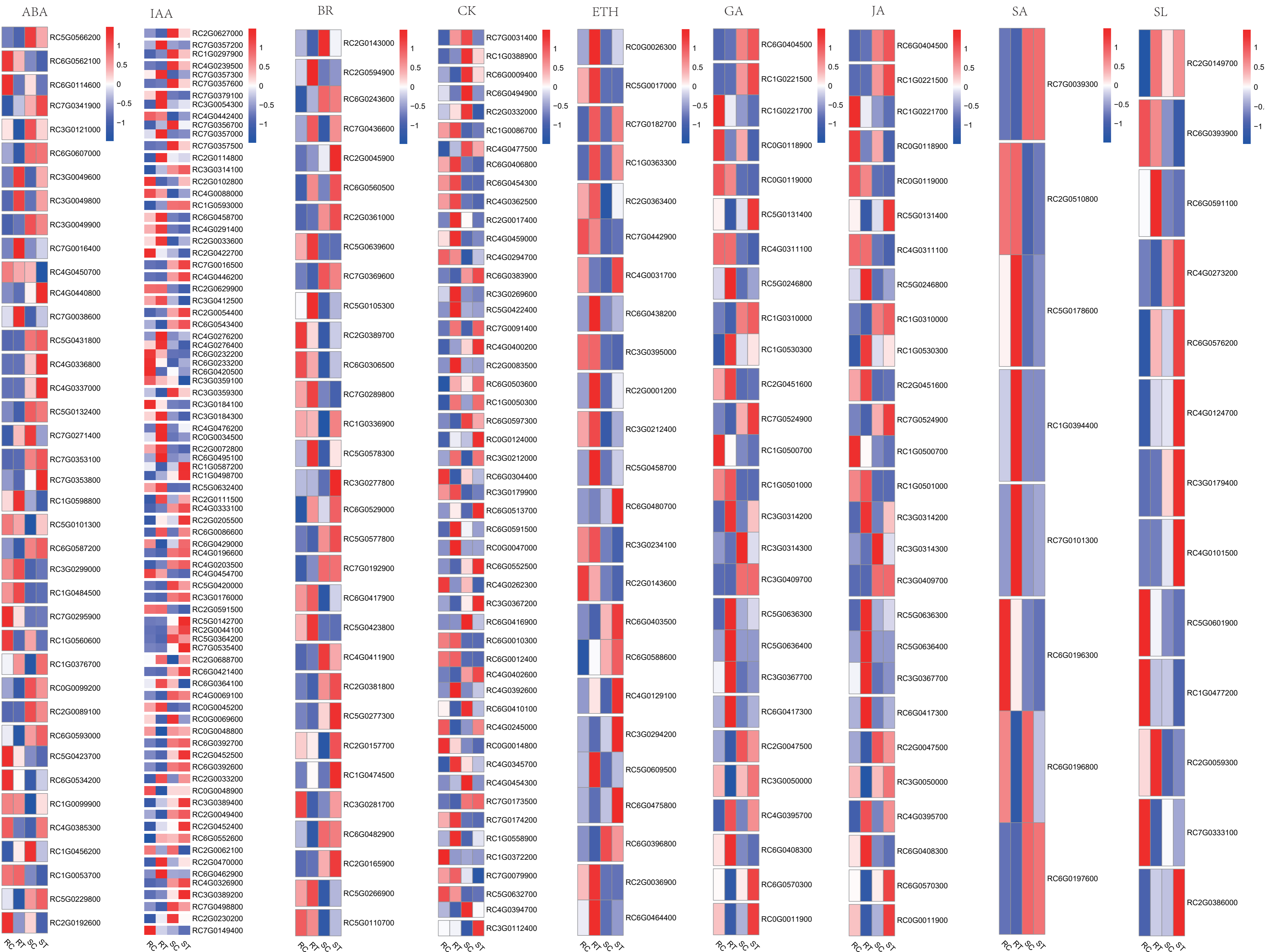

Figure S5. Heatmap showing expression of genes related with phytohormone discovered by mapman.

Supplement: Supplementary file 5 — Supplementary Material 5. [file 12864_2024_10100_MOESM5_ESM.pdf]
