# Supplementary material for: Evaluation of aphid resistance on different rose cultivars and transcriptome analysis in response to aphid infestation
Source: BMC Genomics. 2024 Mar 4;25:232. doi: 10.1186/s12864-024-10100-z (PMC10910744; doi:10.1186/s12864-024-10100-z)
Supplement: Supplementary file 6 — Supplementary Material 6. [file 12864_2024_10100_MOESM6_ESM.pdf]

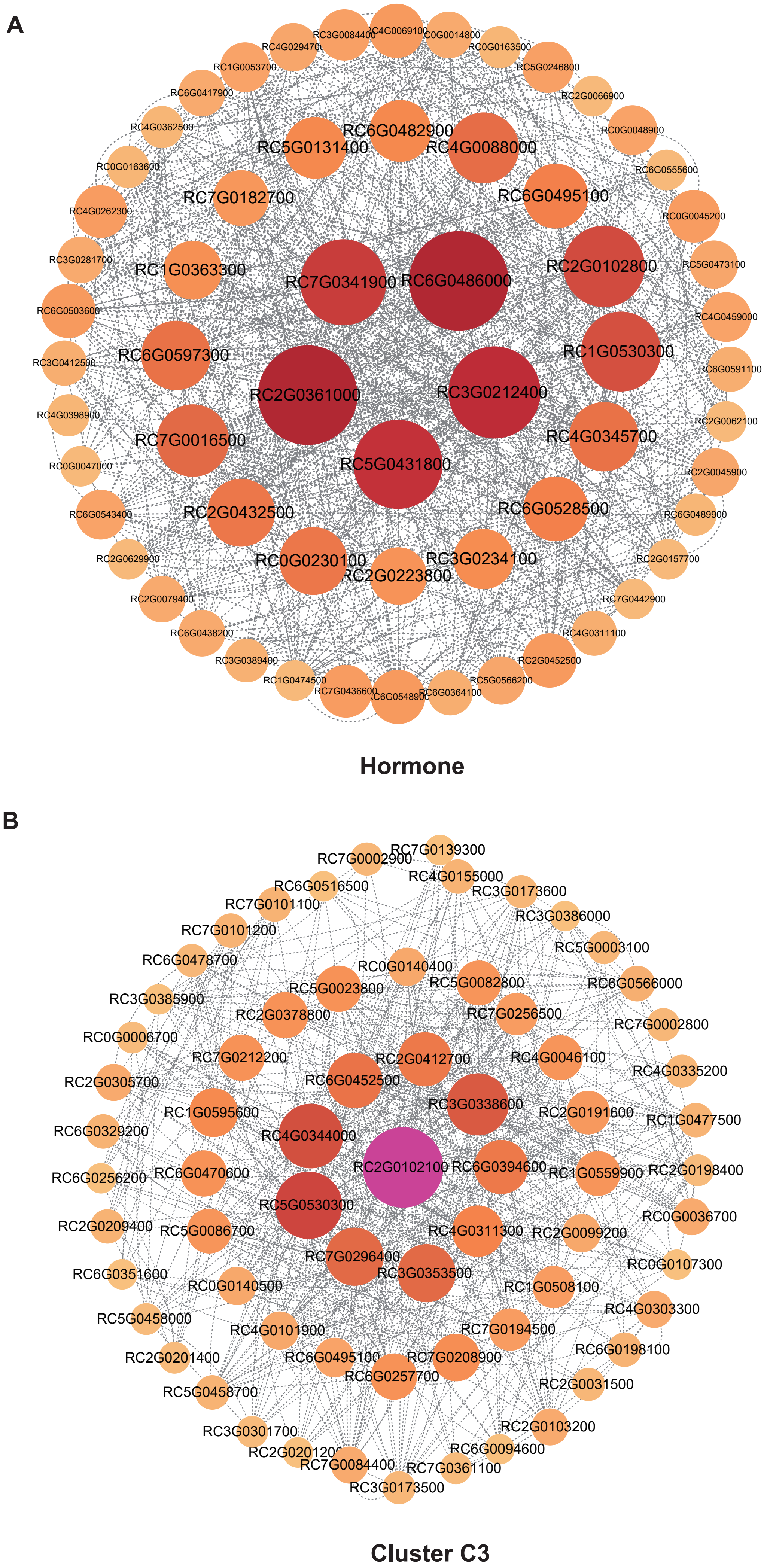

Figure S6. PPI analysis constructed by STRING.
(A) Genes related to hormone metabolism. (B) Genes in cluster C3. Color and node size represent the degree of physical interactions.
